# Haloarchaea Endowed with Phosphorus Solubilization Attribute Implicated in Phosphorus Cycle

**DOI:** 10.1038/srep12293

**Published:** 2015-07-28

**Authors:** Ajar Nath Yadav, Divya Sharma, Sneha Gulati, Surender Singh, Rinku Dey, Kamal Krishna Pal, Rajeev Kaushik, Anil Kumar Saxena

**Affiliations:** 1 Division of Microbiology, Indian Agricultural Research Institute, New Delhi 110012, India; 2 Directorate of Groundnut Research, Junagadh 362001, India

## Abstract

Archaea are unique microorganisms that are present in ecological niches of high temperature, pH and salinity. A total of 157 archaea were obtained from thirteen sediment, water and rhizospheric soil samples collected from Rann of Kutch, Gujarat, India. With an aim to screen phosphate solubilizing archaea, a new medium was designed as Haloarchaea P Solubilization (HPS) medium. The medium supported the growth and P solubilization activity of archaea. Employing the HPS medium, twenty isolates showed the P-solubilization. Phosphate solubilizing archaea were identified as seventeen distinct species of eleven genera namely *Haloarcula, Halobacterium, Halococcus, Haloferax, Halolamina, Halosarcina, Halostagnicola, Haloterrigena, Natrialba*, *Natrinema* and *Natronoarchaeum*. *Natrinema* sp. strain IARI-WRAB2 was identified as the most efficient P-solubilizer (134.61 mg/L) followed by *Halococcus hamelinensis* strain IARI-SNS2 (112.56 mg/L). HPLC analysis detected seven different kinds of organic acids, namely: gluconic acid, citric acid, formic acid, fumaric acid succinic acid, propionic acid and tartaric acid from the cultures of these isolates. These phosphate solubilizing halophilic archaea may play a role in P nutrition to vegetation growing in these hypersaline soils. This is the first report for these haloarchaea to solubilize considerable amount of P by production of organic acids and lowering of pH.

Halophilic archaea (haloarchaea) thrive in environments with salt concentrations approaching saturation^1^,^2^. Many species of haloarchaea of halobacteriaceae family have been isolated from hypersaline environments including *Haloarcula argentinensis*^3^*, Halobacterium* sp.^4^, *Halococcus hamelinensis*^5^, *Haloferax alexandrines*^6^, *Haloferax larsenii*^7^, *Haloferax volcanii*^8^, *Halolamina pelagic*^9^, *Halostagnicola kamekurae*^10^, *Haloterrigena thermotolerans*^11^, *Natrinema* sp.^12^ and *Natronoarchaeum mannanilyticum*^13^. Some of these haloarchaea have been isolated from the rhizosphere of plant species growing in hypersaline environments^14^. Despite the fact that archaea was recognized as a separate kingdom more than 30 years ago, very little is known about their activities in most ecosystems. They are significant contributors to the global carbon and nitrogen cycles; still their involvement in these processes in terrestrial environments is to a large extent unknown. Likewise there are very few reports on characterisation of these halophilic archaea for their plant growth promoting traits so as to help the vegetation to survive better in these extreme environments characterized by nutrient deficient milieu. Soil microorganisms play an important role in soil processes that determine plant productivity. Plant growth promoting (PGP) bacteria may promote growth directly, e.g. by fixation of atmospheric nitrogen, solubilization of minerals to release phosphorus, production of siderophores or 1-aminocyclopropane-1-carboxylate (ACC) deaminase or production of plant growth regulators^15^. Plant growth promoting microbes (PGPM) can have an impact on plant growth providing the plant with a compound that is synthesized by the microbe or facilitating the uptake of nutrients from the environment^16^. Large numbers of eubacteria and fungi have been reported as PGPM but few reports are available for archaea for plant growth promotion which includes nitrogen fixation by methanogens^17^, siderophore production^18^ and Indole-3-acetic acid (IAA) production^19^. However there are no reports available on the ability of archaea to solubilize inorganic and/or organic P from soil.

Different mechanisms have been proposed for solubilization of P from insoluble sources by the action of microorganisms mainly eubacteria and fungi. The major mechanism of mineral phosphate solubilization by eubacteria and fungi is the production of organic acids^20^,^21^. Among the organic acids produced, gluconic, 2-ketogluconic, citric, oxalic, lactic, isovaleric, succinic, glycolic and acetic acids have been most frequently reported from P- solubilizing bacteria^20^,^22^,^23^. Production of organic acids results in the lowering of pH in the surroundings and many reports suggests a positive correlation between lowering of pH and mineral phosphate solubilization. However the reports where such correlation does not exist, suggests mechanisms different from production of organic acids^23^. Some of the alternate mechanisms suggested are production of chelating compounds^24^, inorganic acids like sulphidric, nitric and carbonic acids^25^,^26^. However no reports are available for the mechanism of P solubilization by archaea.

In general, Pikovskaya medium is used for screening of P solubilizing microorganisms. However efforts to screen halophilic archaea employing Pikovskaya medium amended with 5 or 10% NaCl failed. There was need to design a medium that could support the growth of halophilic archaea and also P solubilization activity. The present investigation is the first attempt to design a medium for screening P solubilizing halophilic archaea and to identify P-solubilizing isolates obtained from rhizospheric and non-rhizospheric soils of Rann of Kutch, Gujarat, India. Further the isolates were also investigated for the organic acids profile to elucidate their role in P solubilization.

## Materials and Methods

### Cultures

A total of 157 isolates obtained from thirteen sediments, water and rhizospheric soil samples collected from four different geographical regions (R_1_:23°30′21′′N: 69°39′69′′E, R_2_:23°48′20′′N: 69°43′75′′E, R_3_: 23°57′69′′N: 69°43′95′′E and R_4_: 23°49′67′′N: 69°31′43′′E) of Rann of Kutch, Gujarat, India were used in the present study. The monocot vegetation in the selected hypersaline regions were mainly Banni grasses which included species of *Dicanthium*, *Sporobolous* and *Cenchrus*. Among dicots, *Suaeda nudiflora* of Chenopodiaceae family, *Abutilon* of Malvaceae family and few cucurbits were dominating^27^. The isolates were maintained on seven different halophilic media (DSMZ-97, DSMZ-823, DSMZ-1184, Halophilic medium, chemically defined medium, complex media and OS media) with NaCl concentrations ranging from 10% to 25% (w/v), which is similar to salinity levels of Rann of Kutch^28^. The pure cultures were maintained at 4 °C as slants and glycerol stocks (20%) at −80 °C for further use. The effect of salinity (0 to 5.48 M NaCl) and pH (3 to 11) on archaeal isolates was studied by observing their growth on respective halophilic growth medium following the procedure described earlier^29^.

### Physico-chemical properties of samples

The pH and electrical conductivity of the samples was recorded at sampling site. Soil samples were analyzed for soil organic carbon and total nitrogen (%) according to methods of Walkley and Black^30^ and Bremmer^31^ respectively. Exchangeable cations (Ca and Mg) were extracted with 1 M ammonium acetate (pH 7.0) and estimated by atomic absorption spectrophotometer^32^. Available phosphorus was determined by the Bray II method^33^.

### Formulation of medium

A medium was formulated that could support the growth and P solubilization activity of all the halophilic archaea. Artificial sea water medium was modified to support P solubilization activity and had the following composition (g/L): 10.0 glucose, 1.0 yeast extract, 5.0 tri-calcium phosphate (TCP) or hydroxyapatite (HA) or rock phosphate [RP, a non-detrital sedimentary rock which contains high amounts of phosphate bearing minerals (P2O5: 32%)], 195.0 NaCl, 35.0 MgCl_2_.6H_2_O, 50.0 MgSO_4_.7H_2_O, 5.0 KCl, 0.5 (NH_4_)_2_SO_4_, 1.0 NaNO_3_, 0.5 CaCl_2_.2H_2_O, 0.05 KH_2_PO_4_, 0.03 NH_4_Cl, traces FeSO_4_.7H_2_O, traces MnSO_4_.7H_2_O, 20 agar. pH was adjusted to 7.4 with 1 M Tris base and autoclaved. Filter-sterilized 8% (w/v) NaHCO_3_ and 25% (w/v) sodium pyruvate solutions were added aseptically to the autoclaved medium. The medium was designated as Haloarchaea P solubilization (HPS) medium.

### Estimation of phosphate solubilization

Haloarchaea were qualitatively screened for phosphate solubilization by spot inoculation of 10 μL of archaeal suspension on newly designed HPS agar plates containing TCP as a source of insoluble P. The plates were incubated at 37 °C for 15 days and observed for formation of halo zones around the colonies. The size of phosphate-solubilizing zone was determined for each colony. The isolates positive for P- solubilization were further selected for quantitative estimation of phosphate solubilization. Three replications were maintained for each treatment and uninoculated media served as control. One millilitre of archaeal suspension (3 × 10^9^ cfu/mL) was inoculated to 50 mL of HPS medium and incubated for 14–21 days, at 37 °C. After 21 days, the culture suspension was centrifuged at 10,000 g for 10 min, and the pH of the supernatant was determined with a pH meter (Systronics system 361, India). The soluble P content in the supernatant was spectrophotometrically estimated by the ascorbic acid method^34^. Out of 20 halophilic P-solubilizing archaea, five isolates efficient for mineral phosphate solubilisation with TCP as insoluble source of P were further screened for their growth and P-solubilization using hydroxyapatite or rock phosphate as two different sources of insoluble phosphorus. Growth curves for the five isolates were developed by inoculating micro-well titre plates having 200 μL of DSMZ-1184 and incubated in a temperature-controlled Automated Microbiology Growth Analysis System (Oy Growth Curve Ab Ltd, Finland). All the isolates were analyzed in triplicates and the optical density was measured at 600 nm at regular intervals.

Time course study was carried out to look for mineral phosphate solubilisation at intervals of 7 days up to 21 days with amendment of TCP or HA or RP (5 g/L) as source of insoluble P to HPS medium following the procedure described above.

### Identification of P solubilizing archaeal isolates

The isolates positive for P solubilization in quantitative assays were identified based on sequencing of 16S rRNA. The archaeal genomic DNA was extracted by the method as described earlier^35^ with minor modification in the protocol. Amplification of 16S rRNA gene was done using archaeal specific primers 27F (5′-TTCCGGTTGATCCYGCCGGA-3′) and 958R (5′-YCCGGCGTTGAMTCCAATT-3′). The PCR amplification was carried out in a 100 μL volume by mixing 50–90 ng template DNA with the polymerase reaction buffer (10X); 100 μM (each) dATP, dCTP, dTTP and dGTP; primers 27F and 958R (100 ng each) and 1.0 U Taq polymerase. The amplification conditions were as follows: initial denaturation of 5 min at 95 °C, followed by 25 cycles of 1 min at 95 °C, 1 min at 50 °C and 2 min at 72 °C, and a final extension period of 10 min at 72 °C. The PCR amplified 16S rDNA were purified by QIA quick PCR product purification kit (Qiagen). PCR products of partial 16S rRNA gene were sequenced with fluorescent terminators (Big Dye, Applied Biosystems) and run in 3130xl Applied Biosystems ABI prism automated DNA sequencer at SCI Genome Chennai, India. 16S rRNA gene sequences were analysed using codon code aligner v.4.0.4. The 16S rRNA gene sequences were aligned to those of closely related bacterial species available at GenBank database using BLASTn program. Archaeal isolates were identified based on percentage of sequence similarity (≥97%) with that of a prototype strain sequence in the GenBank. The phylogenetic tree was constructed on the aligned datasets using the Maximum likelihood (ML) method implemented in the program MEGA 4.0.2^36^. The sequences obtained in this study were submitted to the GenBank database at NCBI and accession numbers assigned were KF650663-65, KF650667, KF650669-75, KF650677, KF650679-82, KF650684-85 and KF650691-92.

### Organic acid profile of P solubilizing strains

The archaeal strains were grown in 50 mL of HPS broth supplemented with 0.5% TCP at 37 °C for 15 days at 150 rpm in an incubator shaker (Kuhner LT-X, Switzerland). Two millilitres of culture broth was centrifuged at 12,000 rpm for 10 min and filtered through 0.22 μm nylon filter. Organic acids were analysed through High Performance Liquid Chromatography (HPLC) (Waters) equipped with 2998 PDA detector and autosampler using RP-18 column (250 mm × 4.6 mm). The detection of eluates was carried out at 210 nm and organic acids were identified based on retention time of respective organic acid. The peak areas obtained for the authentic standards for citric acid, formic acid, fumaric acid, gluconic acid, oxalic acid, propionic acid, succinic acid and tartaric acid (Sigma-Aldrich, USA) were used as reference to quantify organic acids in supernatant. Three injections were made for each sample and the values were presented as the mean of three replicates.

## Result

### Physico-chemical characteristics of samples

Physical and chemical characteristics of the different rhizospheric, non-rhizospheric and water samples varied considerably. The values of pH were highly variable from 7.4 to 9.25 (Table 1). Organic carbon content was higher in region 3 (0.62%) in comparison to other regions. Maximum available phosphorus was recorded for rhizospheric samples as compared to non-rhizospheric and water samples.

### Phosphate solubilization and production of organic acids

**Figure 1:**
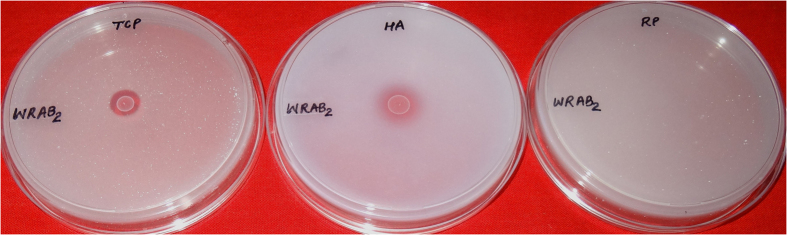
Plates showing zone formation of P-solubilization by archaeal isolates with three different P sources *viz*: tri-calcium phosphate (TCP), hydroxyapatite (HA) and rock phosphate (RP).

The HPS medium designed for studying P solubilization by archaea supported the growth as well as P solubilization activity of halophilic archaea. Among 157 archaeal isolates, twenty exhibited phosphate solubilization both in plates and broth. Clear halo zones were observed around the colonies of isolates that showed P solubilization (Fig. 1). All isolates positive for P solubilization in plate assay also exhibited P solubilization in HPS broth. The ability of archaeal isolates to solubilize P ranged from 10.30 to 134.61 mg/L of P, the maximum being produced by isolate IARI-WRAB2 (Table 2). A significant decline in pH of the culture medium was observed during phosphate solubilization. Isolate, IARI-MAAB1 resulted in significant decline in pH from 7.4 to 3.12 closely followed by IARI-CDK2 where a final pH of 3.18 was achieved after 21 days of incubation (Table 2). All twenty isolates showed variations in their ability to grow at different ranges of pH (4–10) and NaCl concentrations ranging from 1.2–5.48 M. Isolates, IARI-CDK2 and IARI-CSK1 could grow in the range of pH 6–10 while four isolates showed a narrow pH range of 6–8. All the isolates had an absolute requirement for NaCl in the medium for growth (Table 2). In general all the isolates showed growth upto 4.28 M NaCl while seven isolates could grow even beyond.

HPLC analysis of the culture filtrates was performed to identify and quantify the organic acids produced during the solubilization of TCP by 20 archaeal strains. During TCP solubilization all strains showed the production of gluconic acid while none showed production of oxalic acid (Table 3). Quantitative differences in the production of organic acids were observed during the TCP solubilization (Table 3). Only one isolate IARI-SNAB3 showed production of all acids analysed except oxalic acid. The quantities of organic acids produced during TCP solubilization showed significant variations and ranged from 14.63–3533.61 μg/mL for gluconic acid, 44.58–169.24 μg/mL for citric acid, 1178.04–6292.21 μg/mL for formic acid, 0.05–0.39 μg/mL for fumaric acid, 32.74–214.95 μg/mL for propionic acid, 4.01–701.14 μg/mL for succinic acid and 7.64–79.95 μg/mL for tartaric acid (Table 3).

**Figure 2:**
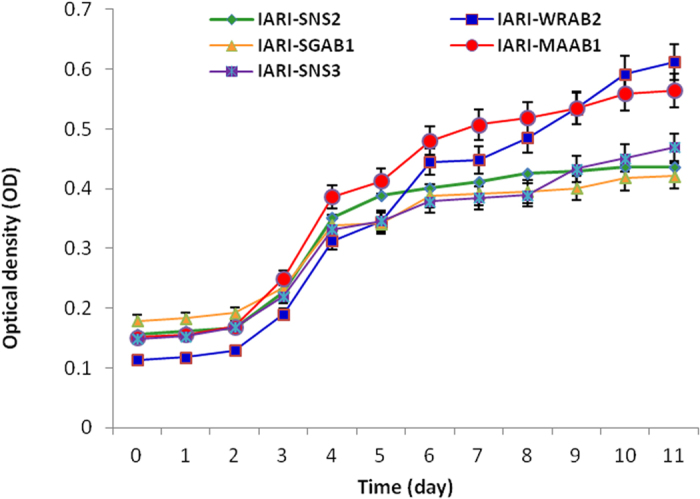
Growth curves of five P-solubilizing archaeal isolates. Bar represents the standard deviation (SD); specific growth rate (0.068, 0.028, 0.029, 0.028 and 0.028) and generation time (13.6, 26.6, 24.3, 25.1 and 25.8) for IARI-SNS2, IARI-WRAB2, IARI-SGAB1, IARI-MAAB1 and IARI-SNS3 respectively.

**Figure 3:**
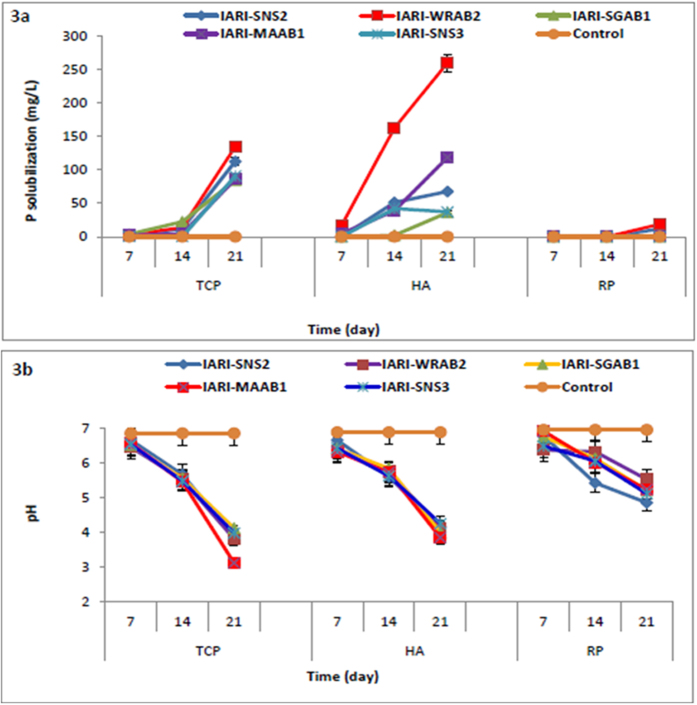
(**a**) P-solubilization by halophilic archaeal isolates in presence of different P sources *viz*: tri-calcium phosphate, hydroxylapatite (HA) and rock phosphate (RP), LSD (P = 0.005) treatments × days: 129.8 for TCP; 1304 for HA and 4.32 for RP. (**b**) Drop in pH during P-solubilization.

Out of 20 archaea, 5 isolates *viz.* IARI-SNS2, IARI-WRAB2, IARI-SGAB1, IARI-MAAB1 and IARI-SNS3 were selected to develop archaeal growth curve and time course study of P solubilization using TCP or HA or RP as source of insoluble phosphate. From the graphical representation of the growth curve presented in Fig. 2, the specific growth rate and generation time for all isolates were extrapolated. All the isolates exhibited a long lag phase of 2 days and reached stationary phase after 10 days of growth. Variations were observed among the isolates with respect to the amount of P-solubilized and the period of incubation. In presence of TCP, three isolates (IARI-WRAB2, IARI-SGAB1, IARI-MAAB1) showed solubilization after 7 days and the values of P solubilized showed increasing trend up to 21 days (Fig. 3a). In case of HA, on 7^th^ d of incubation, only 2 isolates (IARI-WRAB2 and IARI-MAAB1) showed P-solubilization, however, on prolonged incubation for 14 and 21 d all the 5 isolates showed P solubilization activity (Fig. 3a). In presence of RP, however, P solubilization was observed after 21 days of incubation only in treatments inoculated with IARI-SNS2 and IARI-WRAB2 (Fig. 3a). Along with P solubilization, drop in pH of media was observed. Maximum drop of pH from 6.85 to 3.12 was observed in media with TCP followed by 6.88 to 3.85 and 6.95 to 4.85 in media amended with HA and RP respectively (Fig. 3b).

HPLC analysis for type and amount of organic acid revealed variations in both when three different sources of insoluble P were used (Table 3). The comparison of organic acid profiles of two most efficient P- solubilizers, IARI-SNS2 and IARI-WRAB2 revealed that in presence of TCP or HA or RP both produced significantly higher amount of formic acid in comparison to other isolates. The profiles in presence of RP were different as no citric acid was detected and instead propionic acid was produced by these two isolates (Table 3).

### Identification of P solubilizing isolates

**Figure 4:**
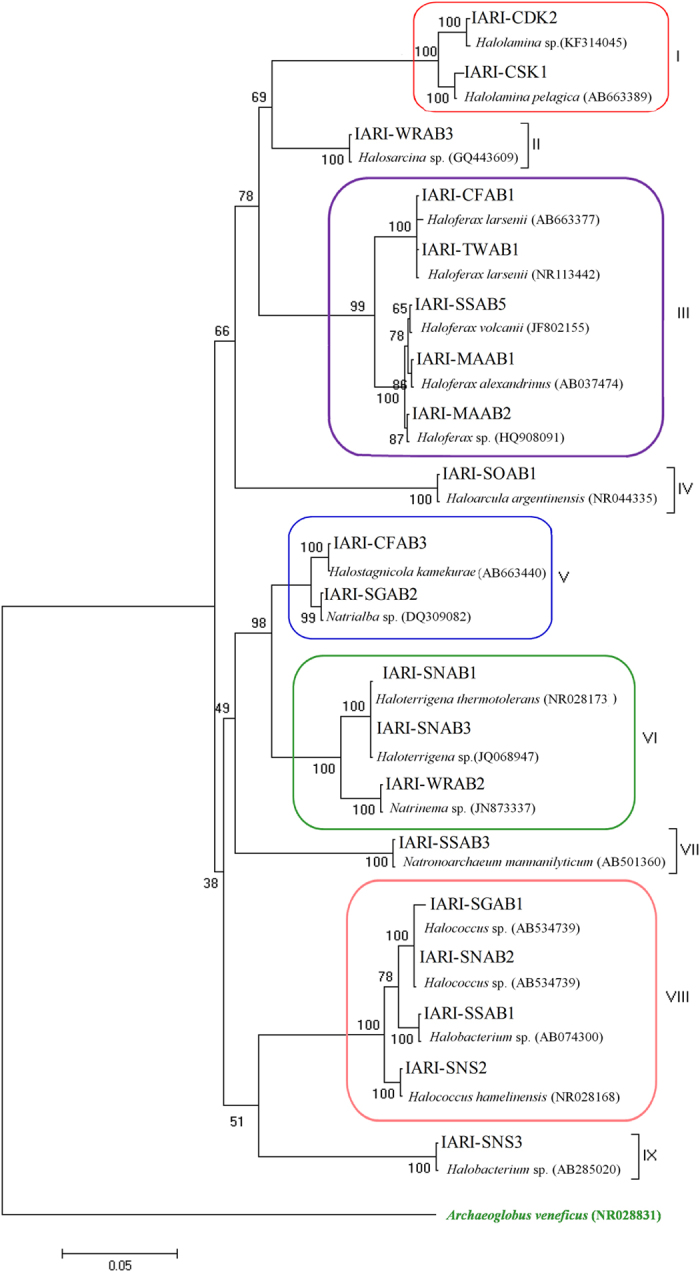
Phylogenetic tree showing the relationships among 20 phosphate solubilizing archaea 16S rRNA gene sequences with reference sequences obtained through BLAST analysis. The sequence alignment was performed using the CLUSTAL W program and trees were constructed using Maximum likelihood (ML) with algorithm using MEGA4 software (Tamura *et al.*^36^). The tree was rooted using *Archaeoglobus veneficus* (NR028831) as the outgroup.

Sequencing of 16S rRNA gene was carried out for the twenty phosphate solubilizing archaea and the sequence data were analysed by BLAST. The nearest match from the NCBI GenBank database for each of the 20 isolates has been reported (Table 2). A phylogenetic tree was constructed using these 20 isolates along with the closest sequences in the NCBI Genbank (Fig. 4). Based on the BLAST analysis, 20 isolates were identified as eleven different genera with seventeen distinct species namely *Haloarcula argentinensis, Halobacterium* sp., *Halococcus hamelinensis, Halococcus* sp., *Haloferax alexandrinus, Haloferax larsenii*, *Haloferax* sp., *Haloferax volcanii, Halolamina pelagic*, *Halolamina* sp., *Halosarcina* sp., *Halostagnicola kamekurae, Haloterrigena* sp., *Haloterrigena thermotolerans, Natrialba* sp., *Natrinema* sp. and *Natronoarchaeum mannanilyticum.* On phylogenetic analysis the 20 phosphate solubilizing archaea were distributed into nine groups (I-IX) as shown in Fig. 4.

## Discussion

Archaea are known to inhabit extreme environments and have never been studied with a perspective to understand their interactions with eubacteria and to sustain vegetation in extremes of salinity, moisture stress and temperature. In the domain of agriculture, archaea have been implicated in nitrogen cycle particularly ammonia oxidation and global methane cycle^37^,^38^. Their role in other nutrient cycles particularly phosphorus is not known. In the present investigation attempt has been made to isolate archaea from hypersaline environment of Rann of Kutch, India and to characterize them for their phosphorus solubilizing capability. P-solubilization is one of the major attribute through which many of the plant growth promoting eubacteria directly influence the growth of plants. Generally, promising P-solubilizing bacteria can be selected on the basis of plate assay wherein insoluble P is solubilized which results in the zone of solubilization. Growth media such as Pikovskaya and NBRIP are reported for screening of solubilizing eubacteria^39^,^40^. However these media were not efficient for the screening of halophilic archaea as their growth requirements are different and have an absolute requirement for NaCl (2–5 M). It is because their proteins remain stable and function in the presence of high salt concentrations^41^. The new medium was formulated that contained higher amounts of MgCl_2_.6H_2_O, MgSO_4_.7H_2_O, KCl and NaCl (35, 50, 5 and 195 g/L as against 0, 0.1, 0.2 and 0 g/L respectively in Pikovskaya medium). In addition the new medium contained sodium pyruvate that supported both growth and P-solubilization activity. It has been reported that critical K concentration is required for optimum P solubilization^23^. In some fungi, Mg and Na have also been implicated in solubilization of P^42^. The ability to solubilize mineral phosphate with TCP as a source is not prevalent among archaea as only 12% of the isolates exhibited this trait. The ability to solubilize P was also influenced by the source of insoluble P used in the medium. All the five archaeal isolates, except IARI-WRAB2 solubilised P in increasing order of RP<HA<TCP. Similar results have been reported for many of the eubacterial isolates like *Pseudomonas striata*, *Bacillus polymyxa* and *Bacillus megaterium*^43^. Isolate IARI-WRAB2 exhibited higher P solubilization in presence of HA as compared to TCP and RP. Such behaviour has also been observed for some of the eubacteria like *Bacillus circulans* and *Bacillus pulvifaciens*^43^. Among the isolates, only two could solubilize RP to a lesser extent as compared to TCP and HA. It has been concluded earlier that tricalcium phosphate and hydroxyapatite are more degradable substrates than rock phosphate^44^.

Growth curve and solubilization rates were also studied to correlate the stage of growth with solubilization efficiency. The maximum amount of solubilized P was recorded only after 21 days of growth irrespective of the source of insoluble P used. This indicates that the concentration of available P is more when the culture is in the stationary phase. It can be concluded that available P in the medium is a consequence of amount of P solubilized due to production of organic acids and amount of P immobilized in the archaeal cell. It appears that in the stationary phase the immobilization of P in the cell system is less as compared to active exponential phase. The preliminary investigation revealed that mechanism of P solubilization by archaea is parallel to eubacteria and is accompanied by lowering of pH due to production of organic acids. Many of the eubacteria and fungi cause a reduction in the pH of the medium either by secretion of various organic acids or by proton (H^+^) extrusion^45^. Proton transport from the cytoplasm to the outer surfaces of the microbes may take place in exchange for a cation (especially ammonium) or with the help of ATPase (ABC transporter) located in the cell membrane and uses the energy from ATP hydrolysis^46^.

Many of the eubacteria had been shown to produce organic acids like gluconic acid, 2-ketogluconic, citric acid, lactic acid and formic acid^47^. The HPLC studies revealed that archaea also produces organic acids like gluconic, citric, fumaric, succinic, propionic, formic and tartaric acid. However the organic acid profiles varied among the twenty isolates analysed. Like eubacteria, gluconic acid was found to be the major acid produced by all the archaeal isolates^48^. Citric acid appears to be the second most important acid being produced by 15 isolates. Few isolates also produced formic acid (7), propionic acid (12), succinic acid (9) and tartaric acid (7). The amount of propionic, succinic, tartaric and fumaric acids produced is comparatively less than gluconic or citric acid and might not have a significant role in P solubilization. Further, no correlation was observed with regards to amount of gluconic or citric acid produced and the concentration of soluble P in the broth. Asea, Kucey^49^ also did not find any correlation between solubilization of P and amount of organic acids produced. However there are reports correlating the two parameters^50^. The amount of P solubilized by archaeal isolates ranged from 10.30 to 134.61 mg/L. This observation cannot be compared since there are no earlier reports on solubilization of P by archaea. However taking a parallel with eubacteria, the amount solubilized is less or comparable to earlier reports published on diverse groups of eubacteria^51^,^52^,^53^,^54^,^55^. Rodríguez and Fraga^44^ reported 11–156 mg/L solubilized P in cultures of different bacterial species grown on TCP. Another study reported 31.5 to 519.7 mg/L solubilized P in different set of strains, maximum being recorded for *Arthrobacter* sp. (CC-BC03) and minimum by *Gordonia* sp. (CC-BC07)^47^. The ability of haloarchaea to solubilize P widens the diversity of microbes endowed with the capability to solubilize P. Different fungi including yeasts, bacteria, actinomycetes and cyanobacteria have already been reported for P solubilization^44^,^56^,^57^,^58^. The ability of archaea to survive in extreme environments and to solubilize P is all the more significant as these hypersaline habitats are characterized by low nutrient mileu. These haloarchaea could help in sustenance of vegetation in harsh environments besides sustaining soil health.

Halophilic archaea are represented primarily by members of the family Halobacteriaceae, but also include methanogens from the genera *Methanohalophilus* and *Methanohalobium* that occur in the sediments of hypersaline lakes. All phosphate solubilizing archaeal sequences obtained in our study by 16S rDNA amplification from the environment grouped within the Halobacteriaceae. There were significant variations within the P solubilizing strains as they belonged to eleven different genera with seventeen distinct species. This first report on P solubilizing capability of haloarchaea indicates that many more cultivable economically important archaeal stains await discovery and utilization in agriculture and allied sectors.

## Additional Information

**How to cite this article**: Yadav, A. N. *et al.* Haloarchaea Endowed with Phosphorus Solubilization Attribute Implicated in Phosphorus Cycle. *Sci. Rep.* **5**, 12293; doi: 10.1038/srep12293 (2015).

## Figures and Tables

**Table 1:** Physico-chemical properties of samples.

Regions	pH	EC mS/cm	%OC	Avail. N (kg/ha)	Avail. P (kg/ha)	Avail. K (kg/ha)	Exch. Ca (mg/kg)	Exch. Mg (mg/kg)
R1	7.40	24.2	0.59	167	14.7	1086	16.93	10.58
R2	9.25	5.42	0.49	209	3.2	5936	57.06	26.36
R3	8.37	1.79	0.62	136	6.23	362	35.45	10.62
R4	8.95	75.25	0.50	142	14.2	550	21.2	7.02

*EC-electrical conductivity; OC-organic carbon*; Avail-available; N-Nitrogen; P-phosphorus; K-Potassium, Ca- calcium; Mg-Magnesium.

**Table 2:** Identification, characterization and phosphorus solubilization attributes of halophilic archaea.

Strain	Nearest phylogenetic relative	Identity(%)	Phenotypic characteristics		Phosphatesolubilisation* (mg/L)	Final pH
			NaCl (M)tolerance	pHrange		
IARI-CDK2	*Halolamina* sp.	99	1.71–5.13	6–10	12.86 ± 1.14	3.18
IARI-SNS3	*Halobacterium* sp.	98	2.57–4.28	5–9	90.99 ± 0.47	3.98
IARI-SNS2	*Halococcus hamelinensis*	99	1.71–5.13	4–8	112.56 ± 0.93	3.85
IARI-MAAB1	*Haloferax alexandrinus*	99	1.71–5.48	5–8	86.36 ± 1.22	3.12
IARI-WRAB2	*Natrinema* sp.	99	3.42–4.28	6–8	134.61 ± 2.28	3.82
IARI-WRAB3	*Halosarcina* sp.	99	2.57–5.13	6–8	30.15 ± 1.48	3.66
IARI-SNAB1	*Haloterrigena thermotolerans*	99	2.57–4.28	6–8	37.97 ± 0.12	3.98
IARI-SNAB2	*Halococcus* sp.	98	1.71–4.28	5–8	41.84 ± 0.88	4.15
IARI-SNAB3	*Haloterrigena* sp.	98	1.71–4.28	6–8	22.90 ± 3.44	4.22
IARI-MAAB2	*Haloferax* sp.	98	1.20–4.28	6–9	10.30 ± 1.27	3.86
IARI-SOAB1	*Haloarcula argentinensis*	99	1.71–4.28	6–9	16.20 ± 0.35	3.54
IARI-SGAB1	*Halococcus* sp.	98	1.71–4.28	5–8	84.45 ± 1.27	4.12
IARI-SGAB2	*Natrialba* sp.	99	2.57–5.48	7–10	75.12 ± 0.80	3.56
IARI-CSK1	*Halolamina pelagica*	98	1.20–5.13	6–10	34.54 ± 1.58	4.10
IARI-SSAB1	*Halobacterium* sp.	99	2.57–4.28	5–9	23.66 ± 0.74	3.90
IARI-SSAB3	*Natronoarchaeum mannanilyticum*	98	2.57–5.13	6–9	16.02 ± 1.08	3.78
IARI-TWAB1	*Haloferax larsenii*	99	1.20–5.48	6–9	12.92 ± 1.05	3.68
IARI-SSAB5	*Haloferax volcanii*	99	1.20–5.48	6–9	16.28 ± 1.33	3.80
IARI-CFAB1	*Haloferax larsenii*	98	1.20–5.48	6–9	10.66 ± 0.76	4.56
IARI-CFAB3	*Halostagnicola kamekurae*	98	3.42–5.13	7–10	16.88 ± 0.48	3.99

^*^The values of P solubilization represents P solubilized in uninoculated control (0.12 mg/L) subtracted from P solubilized by archaeal isolates.

**Table 3:** Organic acid production by halophilic archaea during phosphate solubilization.

Strain	Organic acid (μg/mL)						
	Gluconic acid	Citric acid	Formic acid	Fumaric acid	Propionic acid	Succinic acid	Tartaric acid
TCP							
IARI-CDK2	113.00 ± 2.2	60.77 ± 1.7	ND	ND	ND	ND	ND
IARI-WRAB3	163.48 ± 0.7	99.99 ± 1.4	ND	ND	ND	320.80 ± 1.6	ND
IARI-SNAB1	18.66 ± 1.7	59.28 ± 1.3	1966.98 ± 2.8	ND	101.39 ± 0.8	28.44 ± 2.0	ND
IARI-SNAB2	125.67 ± 3.2	59.37 ± 0.5	ND	0.29 ± 0.3	ND	ND	ND
IARI-SNAB3	3533.61 ± 3.7	77.59 ± 1.2	6292.21 ± 1.1	0.13 ± 0.1	33.53 ± 0.5	160.34 ± 3.2	79.95 ± 2.9
IARI- MAAB2	93.57 ± 2.0	44.58 ± 2.0	38350.87 ± 2.6	ND	214.95 ± 0.8	701.14 ± 1.4	ND
IARI-SOAB1	54.69 ± 0.4	116.06 ± 1.3	ND	0.06 ± 0.1	19.94 ± 0.8	ND	ND
IARI-SGAB2	379.76 ± 0.3	64.22 ± 1.4	ND	0.39 ± 0.1	34.10 ± 1.2	524.91 ± 1.5	28.20 ± 2.5
IARI- CSK1	171.47 ± 0.7	60.56 ± 2.0	ND	0.05 ± 0.2	ND	24.01 ± 2.5	44.73 ± ± 0.5
IARI-SSAB1	54.63 ± 1.8	ND	ND	ND	32.74 ± 0.3	ND	13.96 ± 0.3
IARI-SSAB3	2114.67 ± ± 0.4	ND	ND	0.28 ± 0.2	ND	ND	ND
IARI- TWAB1	114.82 ± 1.8	ND	ND	ND	ND	ND	11.40 ± 0.6
IARI-SSAB5	1342.56 ± 3.4	108.61 ± 1.9	ND	0.33 ± 0.1	ND	29.09 ± 1.1	ND
IARI-CFAB1	44.86 ± ± 1.6	ND	ND	ND	57.35 ± 0.9	ND	ND
IARI- WRAB2	329.05 ± ± 1.3	ND	ND	0.32 ± 0.3	ND	192.58 ± .6	ND
IARI-SNS2	37.03 ± 1.3	125.09 ± 1.2	4854.53 ± 2.0	ND	ND	26.50 ± 2.1	7.64 ± 1.9
IARI- WRAB2	14.63 ± 1.9	95.58 ± 3.4	2506.19 ± 3.9	0.19 ± 0. 2	43.63 ± 0.5	ND	7.52 ± 2.9
IARI-SGAB1	1182.61 ± 1.9	169.24 ± 1.2	ND	0.07 ± 0.1	80.19 ± 1.1	ND	ND
IARI-MAAB1	34.43 ± 1.4	117.90 ± 1.5	1178.04 ± 2.7	ND	67.07 ± 1.3	ND	ND
IARI-SNS3	120.53 ± 2.0	111.16 ± 1.4	4896.73 ± 1.0	ND	41.29 ± 1.0	ND	ND
HA							
IARI-SNS2	51.98 ± 1.02	72.01 ± 1.35	1313.27 ± 1.03	ND	ND	ND	ND
IARI-WRAB2	113.00 ± 1.13	ND	1694.76 ± 1.34	ND	ND	ND	ND
IARI-SGAB1	316.53 ± 2.08	85.60 ± 2.56	1128.46 ± 2.77	ND	ND	ND	ND
IARI-MAAB1	4944.2 ± 5.26	54.92 ± 1.12	1154.57 ± 1.61	ND	278.4 ± 0.77	ND	ND
IARI-SNS3	4431.5 ± 2.71	45.74 ± 1.37	1114.13 ± 2.23	ND	211.1 ± 1.32	ND	ND
RP							
IARI-SNS2	48.67 ± 1.46	ND	1041.73 ± 1.38	ND	242.5 ± 0.62	ND	ND
IARI-WRAB2	42.45 ± 2.98	ND	962.94 ± 2.28	ND	77.3 ± 0.87	ND	ND
IARI-SGAB1	ND	ND	ND	ND	ND	ND	ND
IARI-MAAB1	ND	ND	ND	ND	ND	ND	ND
IARI-SNS3	ND	ND	ND	ND	ND	ND	ND

Values are the mean of three replicates ± standard error; tri-calcium phosphate (TCP); Hydroxyaptite (HA); Rock phosphate (RP).
